# Polycyclic Aromatic Hydrocarbons in the Estuaries of Two Rivers of the Sea of Japan

**DOI:** 10.3390/ijerph17176019

**Published:** 2020-08-19

**Authors:** Tatiana Chizhova, Yuliya Koudryashova, Natalia Prokuda, Pavel Tishchenko, Kazuichi Hayakawa

**Affiliations:** 1 V.I.Il’ichev Pacific Oceanological Institute FEB RAS, 43 Baltiyskaya Str., Vladivostok 690041, Russia; koudryashova@poi.dvo.ru (Y.K.); tpavel@poi.dvo.ru (P.T.); 2 Institute of Chemistry FEB RAS, 159 Prospect 100-let Vladivostoku, Vladivostok 690022, Russia; nataprokuda@gmail.com; 3 Institute of Nature and Environmental Technology, Kanazawa University, Kakuma, Kanazawa 920-1192, Japan; hayakawa@p.kanazawa-u.ac.jp

**Keywords:** estuarine PAH pollution, seasonal PAH variability, transboundary pollution, riverine PAH mass flux

## Abstract

The seasonal polycyclic aromatic hydrocarbon (PAH) variability was studied in the estuaries of the Partizanskaya River and the Tumen River, the largest transboundary river of the Sea of Japan. The PAH levels were generally low over the year; however, the PAH concentrations increased according to one of two seasonal trends, which were either an increase in PAHs during the cold period, influenced by heating, or a PAH enrichment during the wet period due to higher run-off inputs. The major PAH source was the combustion of fossil fuels and biomass, but a minor input of petrogenic PAHs in some seasons was observed. Higher PAH concentrations were observed in fresh and brackish water compared to the saline waters in the Tumen River estuary, while the PAH concentrations in both types of water were similar in the Partizanskaya River estuary, suggesting different pathways of PAH input into the estuaries. The annual riverine PAH mass flux amounted to 0.028 t/year and 2.5 t/year for the Partizanskaya River and the Tumen River, respectively. The riverine PAH contribution to the coastal water of the Sea of Japan depends on the river discharge rather than the PAH level in the river water.

## 1. Introduction

Polycyclic aromatic hydrocarbons (PAHs) are a class of hydrophobic organic compounds composed of several fused aromatic rings. They are widespread environmental pollutants due to the stability imparted by their lack of functional groups. Furthermore, some PAHs are carcinogenic and toxic as well as bioaccumulative in food chains [1,2,3]. Thus, PAH pollution has the potential to change the structure and dynamics of biological communities, creating environmental risks for ecosystems and subsequently for human well-being.

Significant amounts of PAHs are released into the environment, including the water compartment, since they occur with the burning of fossil fuels, and, moreover, are part of them, and the consumption of fossil fuels globally is high today [4]. The estuarine zones, which are usually densely populated, are under heavy pressure from these pollutants. Furthermore, estuaries are the recipients and transmitters of upstream water basin pollution into the marine environment. Due to the hydrodynamic properties and the existence of strong gradients of chemical parameters, primarily the salinity gradient, estuaries act as a biogeochemical barrier, trapping a portion of the terrigenous material, especially suspended matter, containing anthropogenic organic substances such as PAHs [5]. As a result, estuaries are loaded with PAHs to a greater extent than bordering ecosystems. Therefore, estuarine PAH pollution is a crucial problem that requires knowledge about PAH occurrence and biogeochemical behaviour in this environment to develop effective strategies for pollution control and management.

Estuaries of the Sea of Japan, in particular of its Russian northwestern region, are important to the fisheries industry of countries around the sea (Russia, Japan, South Korea and North Korea). They serve as nursery, feeding and refuge areas for commercially important species of fish such as salmonids and various shellfishes [6], providing a functioning ecosystem in the Sea of Japan. Nevertheless, because the Russian northwestern coast of the Sea of Japan has the highest population density in the Russian Far East, its rivers receive high contributions from municipal and industrial effluents and agricultural run-off [7,8]. Furthermore, due to the fact that some of the rivers are transboundary and collect pollution from the territories of two or more countries, their estuaries can be persistently exposed to significant nutrient, heavy metal and organic pollutant loads [9,10]. Although a number of studies are devoted to the spatial and temporal PAH dynamics in the coastal and open waters of the Sea of Japan [11,12,13,14], data on the PAHs in the estuaries of the rivers that flow into to the Sea of Japan are scarce to date. Moreover, information about seasonal PAH variability in these estuaries is not available. A single PAH survey in a few rivers and estuaries adjacent to the Sea of Japan (the Russian region) found low levels of contamination in the summer [15]. However, given that studies in the Russian coastal waters of the Sea of Japan have shown a significant increase in PAH concentrations during the cold season due to emission from heating systems [16], this assessment may not be correct.

The objective of this study was to assess the estuarine PAH pollution of the two rivers of the Sea of Japan, namely the Tumen river and the Partizanskaya river, taking into account seasonal PAH variability, and to estimate potential environmental risks. Furthermore, since the estuary is formed by sea and river waters, the relationship between PAH and salinity was examined to understand the PAH pathway to the estuary (from the sea or with river runoff) and its influence on PAH dispersion in the estuary system. Additionally, annual and seasonal PAH riverine mass fluxes to the coastal environment were calculated to design the future PAH budget for the Sea of Japan.

## 2. Materials and Methods

### 2.1. Study Area Characteristic

The Tumen river is the largest river in the northwestern Sea of Japan, with a catchment area of 41,200 km^2^. China owns 70% of the river’s catchment; almost 30% is owned by North Korea, and Russia’s share in the downstream catchment is less than 1%. According to data from the early 2000s, the population of the river basin area was 2.2 million people. The Tumen River supplies water for human consumption, agricultural irrigation and industrial development in China and North Korea. Its source is located on the Changbai Mountain Plateau in the DPRK, and a significant part of the river’s length is in the middle between the North Korean and the East Manchurian Mountains, forming the border between the DPRK and China. In the lower reaches, the river forms part of the border between the DPRK and Russia. The Tumen River eventually flows into Peter the Great Bay (the northwestern Sea of Japan) as two water streams. One is a main water stream 600 m wide and another is much smaller, periodically overlapping a sand drift. In the main water stream, the sea-water intrusion occurs from the river bar into the upstream river over 3 km. The discharge volume rate of the Tumen River is about 200 m^3^/s (in May and October) [17].

The Partizanskaya River, the catchment of which is located entirely within the territory of Russia, is of great importance for Russian agricultural development and fisheries. Its length is 142 km, and the catchment area is 4140 km^2^. The river flows into Peter the Great Bay, and it has a single riverbed, which is up to 200 m wide. The sea-water intrusion in the estuary depends on the river discharge volume rate and varies between 5 and 12.5 km, with a discharge volume rate ranging from 70 to 7.8 m^3^/s [18]. Since the maximum tidal range is 40–50 cm in Peter the Great Bay, the estuaries of both rivers under study are classified as microtidal or highly stratified [17,18].

The study area is under the influence of a temperate monsoon climate, with winter lasting from mid-November to March. The average temperature is −8 °C to −14 °C in winter and +17 to +22 °C in summer. The rivers freeze solid at the end of November, while final ice removal occurs at the end of March. Due to the monsoon climate, the Tumen river and the Partizanskaya river discharge depends strongly on the season. The characteristic features of the summer are heavy rainfall and fog; furthermore, tropical typhoons that arise over the Pacific Ocean are regular occurrences in this season. As a result, the maximum discharge is usually observed in summer.

### 2.2. Sampling Points and Sample Collection

**Figure 1:**
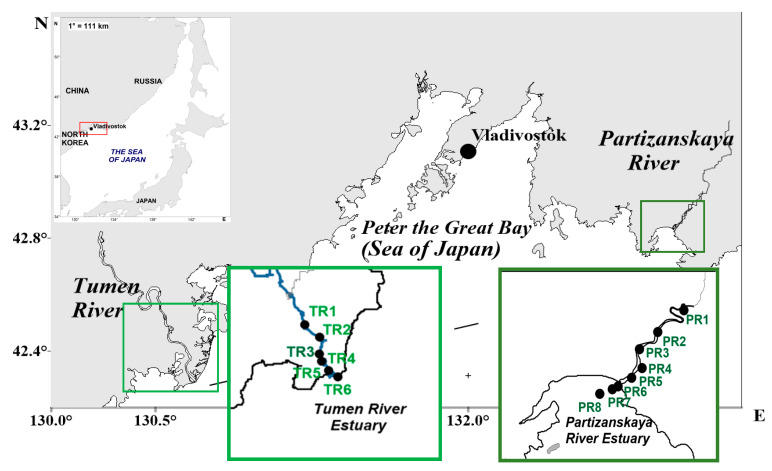
Location of the sampling stations in the Tumen River Estuary and the Partizanskaya River Estuary. The water samples were collected at stations TR1–TR5 in winter, TR2–TR6 in summer and TR1–TR6 in spring and autumn in the Tumen River Estuary.

To study the seasonal variability of PAHs, four sampling campaigns during the year were conducted in January, May, July and September 2012 for the Partizanskaya River estuary and in February, April, July and October 2017 for the Tumen River estuary. Water samples were collected at eight stations in every season for the Partizanskaya estuary and at five and six stations in every season for the Tumen River estuary (Figure 1). At each station, one surface water sample (about 0.5 m under the surface) and one bottom water sample (about 0.5 m from the bottom) were collected in every season except winter in the Partizanskaya estuary, when only surface (under ice) water samples were collected. The depths of the sampling sites were 1.5–10 m and 1.5–42 m for the Partizanskaya River and the Tumen River estuaries, respectively. Totals of 56 and 44 water samples were collected in the Partizanskaya estuary and the Tumen estuary, respectively.

Five litres of estuarine water were collected at every sampling point in Niskin bottles directly from the ice cover in winter and from the rubber boat in other seasons. The vertical profiles of temperature, conductivity (salinity), dissolved oxygen and turbidity were analysed using an RBR-XRX620 sensor (RBR Ltd., Ottawa, ON, Canada).

### 2.3. Sample Pre-Treatment and Analysis

To separate PAHs contained in dissolved and particulate phase (DPAHs and PPAHs), water samples (5 L) were filtrated through a fibreglass filter (pore size 0.5 μm, Advantec GC50, Japan). DPAHs from the remaining water were concentrated using solid-phase extraction on C-18 cartridges (Waters Sep-Pak C-18, Cartridge, UK). Before extraction, SPE cartridges were preconditioned by 5 mL of methanol followed by 5 mL of distilled water. Then, the water that contained DPAHs was passed through SPE cartridges with a flow rate of approximately 10 mL/min. The filters and the cartridges were stored at −20 °C until high-performance liquid chromatography (HPLC) analysis.

Prior to HPLC analysis, 20 μL of an internal standard containing naphthalene-*d8*, acenaphthene-*d10*, phenanthrene-*d10*, pyrene-*d10* and benzo[*a*]pyrene-*d12* was added to the SPE cartridges and the filters. PAHs were eluted from the filters and SPE cartridges by different methods. The DPAHs were desorbed from the SPE cartridges using 15 mL of dichloromethane. The solution was concentrated by rotary evaporation and the DPAHs that remained were dissolved in 1 mL of hexane for cleaning up on silica gel cartridges (Water Sep-Pak Silica, Cartridge, UK). First, the silica cartridges were preconditioned by 5 mL of hexane, then the extracts were applied to the cartridges. The DPAHs were eluted from the cartridges by 15 mL of hexane in acetone (9:1, *v*/*v*), and 200 μL of dimethylsulfoxide (DMSO) was added to the solution. The solvent was removed by rotary evaporation until only DMSO and the residue was dissolved in 800 μL of acetonitrile for the HPLC analysis.

The filters containing PPAHs were ultrasonicated twice by 40 mL of benzene in ethanol (3:1, *v*/*v*). The combined solution was extracted by liquid–liquid extraction with 80 mL of sodium hydroxide solution (5%), 80 mL of sulfuric acid solution (20%) and 80 mL of Milli-Q water (performed twice). The DMSO (200 μL) was added to the solution, and the solvent was removed by rotary evaporation. The remaining DMSO was reconstituted to 1 mL in acetonitrile for HPLC analysis.

The determination of PAHs was conducted using HPLC equipment with a fluorescent detector (L series, Hitachi High Technologies, Tokyo, Japan). An Inertsil ODS-P analytical column (250 × 4.6 mm, 5 µm) and an injection volume of 20 µL were employed. The temperature of the column was kept constant at 20 °C. The mobile phase was a mixture of acetonitrile–water delivered in gradient mode with acetonitrile increasing from 55 to 100% over 60 min. The flow rate of the mobile phase was maintained at 1 mL/min. Both the excitation and emission wavelengths of the fluorescence detector were set at optimum wavelengths for each PAH with a time program.

A total of 13 PAHs from the USEPA’s 16 priority PAHs list were quantified: the 3-ring PAHs were acenaphthene (Ace), fluorene (Fle) and anthracene (Ant); the 4-ring PAHs were fluoranthene (Flu), pyrene (Pyr), benz[a]anthracene (BaA) and chrysene (Chr); the 5-ring PAHs were benzo[b]fluoranthene (BbF), benzo[k]fluoranthene (BkF), benzo[a]pyrene (BaP) and dibenz[a,h]anthracene (DBA); the 6-ring PAHs were benzo[g,h,i]perylene (BPe) and indeno [1,2,3-cd]pyrene (IDP). The analytical data of Nap and Phe were not reported due to the low recovery of Nap in the particulate phase and the imperfect resolution of Phe with interfering peaks for the dissolved phase. In addition, acenaphthylene does not fluoresce and was thus also excluded from analysis. Three-ring compounds are low molecular weight (LMW) PAHs and five- to six-ring compounds are high molecular weight (HMW) PAHs.

### 2.4. QA/QC

The limit of detection (LOD) and limit of quantification (LOQ) were evaluated based on signal to noise ratio cut-offs of 3 and 10, respectively. The LOD ranged between 0.04 pg/injection (Ant) and 2.86 pg/injection (IDP). The LOQ varied between 6 ng/L (Ant) and 477 ng/L (IDP).

To detect the contamination due to sampling and pre-treatment procedures, two field blanks were performed for every seasonal sampling; in addition, two laboratory blanks for each set of estuarine samples were prepared and treated. Average field blank data ranged from below LOD to 0.2 ng/L (Ace), and the PAHs with concentrations higher than the LOQs were subtracted from the related PAH concentrations in the samples; laboratory blank data were below the LOD and LOQ data and were not taken into account.

The internal standard procedure was used for quantitation. Recoveries of PAH internal standards were 64.9 ± 7.43 and 85.4 ± 7.41 for Ace-*d*_10_, 68.9 ± 9.18 and 84.2 ± 7.17 for Phe-*d*_10_, 80.6 ± 10.24 and 83.8 ± 9.83 for Pyr-*d*_10_, 104.6 ± 12.35 and 101.6 ± 8.40 for BaP-*d*_12_ for dissolved and particulate samples, respectively (*n* = 100 for each phase).

### 2.5. Ecological Risk Assessment

Examination of potential environmental risk of PAHs for the estuarine-coastal ecosystems was carried out by using risk coefficient RQ [19].

RQ = C_PAHs_/C_QV_ (1)

where C_PAHs_ is the concentration of an individual PAH, C_QV_ is the quality values for each PAH. Based on QV such as the negligible concentrations (NCs) and the maximum permissible concentrations (MPCs) of PAHs taken from [19], the following risk coefficients were calculated:

RQ_NCs_ = C_PAHs_/C_QV(NCs)_ (2)

RQ_MPCs_ = C_PAHs_/C_QV(MPCs)_ (3)

where C_QV(NCs)_ was the quality values of the NCs of PAHs and C_QV(MPCs)_ was the quality values of the MPCs of PAHs (the quality values are presented in Table S1). Levels at RQ_NCs_ < 1 and RQ_MPCs_ < 1 suggest low eco-toxicological risks, while levels at RQ_NCs_ > 1 and RQ_MPCs_ < 1 suggest moderate risks, and RQ_NCs_ > 1 and RQ_MPCs_ > 1 show high risks.

### 2.6. Mass Fluxes Calculation

The seasonal and annual river PAH mass flux was estimated based on the daily PAH flux (J_i_) calculated using the following formula [10]:

J_i_ = Q * C_i_ (4)

where Q is the daily discharge of the river taken in [20], m^3^/s; C_i_—daily concentration of TPAH (a sum of DPAHs and PPAHs). The daily TPAH concentration in the estuaries, based on the average values of surface and bottom TPAHs, was calculated using the equation of a linear relationship between PAH concentrations and river discharge on sampling days (Figure S1).

### 2.7. Data Analysis

Univariate statistical analyses were performed with STATISTICA Software (Version 10, StatSoft Inc., Tulsa, OK, USA).

Principal component analysis (PCA) is a multivariate statistical method for examining factors to reveal relationships and patterns within datasets, and it has been used to identify PAH origins [21,22]. PCA was performed using MATLAB R2015b for surface water samples from each of the rivers studied. Data submitted to the analysis were arranged in a matrix composed of 13 variables (PAH compounds) and the appropriate number of sample sites. The size of the matrix was 13 × 32 for the Partizanskaya River and 13 × 22 for the Tumen River. Prior to PCA, the original dataset of PAH concentrations was standardized by scaling the values to the mean and standard deviation. The number of factors extracted was dictated by eigenvalues being greater than 1. The results of the PCA are presented by loading and score plots.

## 3. Results and Discussion

### 3.1. PAH Levels in the Tumen and Partizanskaya River Estuaries

The average annual concentration of TPAHs was 33.3 ± 35.1 ng/L in the Tumen River estuary and 20.3 ± 10.6 ng/L in the Partizanskaya River estuary (Table 1). Table 1 summarizes the PAH concentrations reported for selected estuaries worldwide over the past decade. According to the table, the average annual PAH concentrations found in both estuaries were in the low ng/L range. However, there have been seasonal increases in the PAH level, which will be discussed below. A comparison of the levels of the same PAHs in the estuaries under study and in the rivers presented in Table 1 and in work [23] showed that the average annual PAH concentrations found in both estuaries were in the low ng/L range.

The average annual concentrations of DPAHs and PPAHs were 17.6 × 13.8 and 15.7 × 21.7 ng/L in the Tumen river estuary and 15.4 × 9.5 and 5.3 × 2.6 ng/L in the Partizanskaya river estuary (Table 1). No statistically significant differences were found between the dissolved and particulate PAH contents in the estuarine waters of both rivers. This is consistent with results previously obtained in rivers and estuaries of the northwestern Sea of Japan [15] and contrary to the data from PAH studies in the coastal environment of the Sea of Japan, demonstrating that the DPAH fraction is larger than the PPAH fraction [16,24,25]. This finding indicates that the estuaries under study are effective geochemical barriers where a significant amount of PPAHs are deposited.

In the Tumen River, the PAH concentrations in the estuarine samples were below those in the middle river reach samples, with mean annual PAH concentrations of 68 and 104 ng/L re-estimated for the 13 researched DPAHs and PPAHs, respectively [21]. Apparently, the higher PAH concentration in the middle Tumen River results from severe anthropogenic stress from Chinese and North Korean industrial and residential activities [31], while surrounding estuary areas are scarcely populated and have no relevant roads or industries. Thus, the PAHs observed in the estuary were certainly caused by transboundary PAH transport from polluted upstream areas.

### 3.2. Seasonal PAH Variability

**Figure 2:**
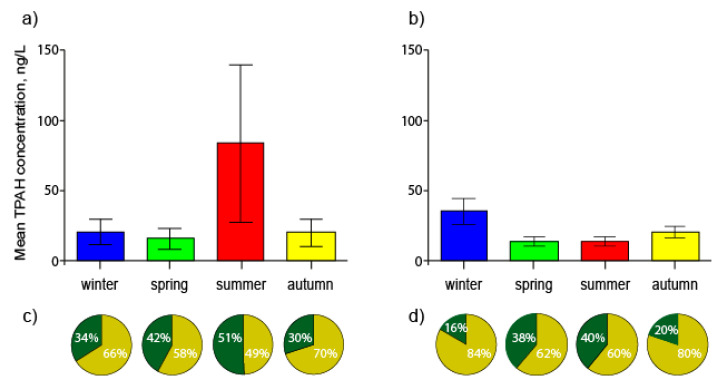
The seasonal variations of TPAHs in the estuarine waters of the Tumen River Estuary (**a**) and the Partizanskaya River Estuary (**b**) and relative contribution (%) of dissolved and particulate PAHs in the Tumen River Estuary (**c**) and the Partizanskaya River Estuary (**d**). Error bars represent one standard deviation from the mean value. The Tumen River Estuary: *n* = 10 in winter and summer, *n* = 12 in spring and autumn. The Partizanskaya River Estuary: *n* = 8 in winter, *n* = 16 in every other season.

The mean seasonal TPAHs concentration of the Tumen River varied from 15.6 to 83.2 ng/L over the year, with the highest concentrations occurring in the summer (Figure 2a). The mean seasonal TPAHs in the Partizanskaya river estuary varied from 13.3 to 35 ng/L and demonstrated the opposite trend to TPAHs in the Tumen River estuary, increasing from autumn and reaching a maximum in winter. As can be seen, in general, the PAH level in the estuaries studied was low during the year; however, in the summer the average TPAH concentration in the Tumen River reached a moderate level of PAH pollution on a global scale [23].

In the Partizanskaya river estuary, the trend in seasonal PAH variation is typical for regions under climatic conditions with a cold season and is described in a number of works, including for coastal water of the northwestern Sea of Japan [16,27,31]. The noticeable rise in TPAHs concentrations observed during the cold period resulted from significant PAH emissions from residential and municipal heating systems.

In the Tumen River, the seasonal PAH variability is similar to the trend found for some water bodies located at latitudes similar to the areas under study. An increase in TPAHs was found in the warm, wet period, explained by a higher run-off PAH input due to heavy precipitation, as well as greater solubility of PAHs at elevated temperatures [32,33]. Accordingly, in the summer, when the greatest amount of precipitation was recorded, the highest TPAHs were found for the Tumen river.

Furthermore, it has been shown that PAHs in river systems can be derived from soil pollution [34]. Therefore, soil particles with PAHs could have been washed away and entered the riverine water body from the entire drainage area that is severely polluted in the middle part, thereby increasing PAHs in the estuary. Indeed, in the summer, an increase in the proportion of PPAHs from TPAHs was observed, reaching 51% (on average) versus 30%, 34% and 42% in the autumn, winter and spring, respectively (Figure 2b). It should be noted that in the Partizanskaya River estuary, the proportion of PPAHs from TPAHs was 38% and 40% in the spring and summer versus 16% and 20% in the winter and autumn, respectively, which also indicated the potential contribution of soil particles containing PAHs. However, TPAHs were observed to be larger in the winter, more than double compared with other periods, as discussed above. These findings are similar to those found for the middle Tumen River [21] and demonstrate that the seasonal change in PAHs driven by heating was revealed in the areas experiencing anthropogenic loading and was not noticeable in remote areas where the PAH enrichment in the river environment was mainly due to seasonal natural factors—for example, rainfall run-off.

### 3.3. Compositional PAH Profiles

**Figure 3:**
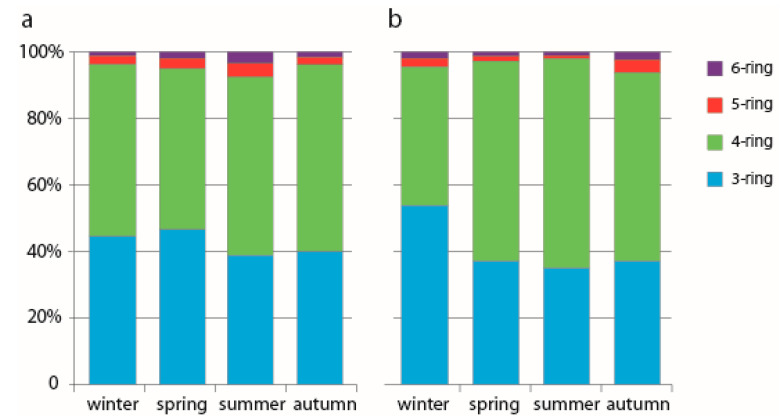
The percent composition of 3-, 4-, 5-, and 6-ring TPAHs in different seasons in waters of the Tumen River estuary (**a**) and the Partizanskaya River estuary (**b**).

The relative TPAH concentrations in the estuaries of the Tumen and Partizanskaya rivers are shown in Figure 3a,b. In the TPAH compositional profile, 3- and 4-ring PAHs accounted for the largest part, with 4-ring PAHs dominating except for winter data for the Partizanskaya River. The predominance of 4-ring PAHs over 3-ring ones (studied in this work) was also found for the Jiulong River and the Tiber River estuaries [35,36] and may be associated with the high estuarine bioproductivity. As estuarine ecosystems are extremely productive, it is possible that the higher biodegradation rates of less stable 3-ring PAHs can lead to their depletion in estuaries.

For both estuaries, the relative PAH contents in the dissolved and particulate phases were similar (Figure S2). PAHs with three rings were predominant in the dissolved phase, while 4-ring PAHs prevailed in the particles. The proportion of PAHs with 5–6 rings was higher in the particulate phase than in the dissolved one (except autumn data in the Partizanskaya river estuary), which is consistent with the hydrophobicity of these compounds. Moreover, the dominant individual PAHs, being the same for both estuaries, were as follows: Fle (31–50%), Pyr (11–25%), Ace (11–23%) and Flu (10–20%) in the dissolved phase and Pyr (25–56%), Flu (21–44%), Fle (6–13%) and Chr (3–11%) in the particulate one.

There was a decrease in the share of PAHs with three rings in the summer, which was less pronounced in the Tumen River and stronger in the Partizanskaya River. This can be attributed to changes in hydrological and biogeochemical conditions due to summer river flood. With increasing river discharge, an increased concentration of trace metals in many rivers, including rivers of the Northwestern Sea of Japan, was observed [37,38]. Some trace metals enhance the photodegradation of 3-ring PAHs several times in relation to LMW PAHs or they inhibit the photodegradation of HMW PAHs [39,40]. Moreover, flood events alter the activity of microbial communities [41], and the shift in the summer PAH compositional profile may be associated with higher microbial degradation of the LMW PAHs in the estuaries. Furthermore, in general, PAHs with 2–3 rings are regarded as characterizing petrogenic sources, while the larger 4–6 ring PAHs are characteristic of pyrolytic sources [42]. Therefore, in the Partizanskaya River estuary, the significant relative content of PAHs with three rings in the winter suggests that the river is heavily loaded with PAHs of petrogenic origin, probably washed out from coal dust, which was observed to be spread on the river snow–ice cover.

Seasonal variability in the proportion of HMW PAHs demonstrated that in the Partizanskaya river estuary, the highest relative contribution of 5–6-ring PAHs was in the autumn. This suggests PAH input from pyrogenic sources. As for the Tumen river estuary, higher 5–6-ring PAHs were observed in summer. In this period, PAH pollution of the Tumen river estuary is heavily influenced by the input of surface run-off with PAHs stored for a time in the soil. Probably, HMW PAHs were degraded more slowly than others, and, therefore, as run-off was magnified in the summer, the fraction of 5–6-ring PAHs may become prominent in other seasons.

### 3.4. PAH Source Apportionment

To identify the origin of PAHs in the estuaries, we used Flu/(Flu + Pyr) and IDP/(IDP + BPe) ratios since these ratios are sufficiently conservative, according to [43]. Nevertheless, PAH ratios should be applied with caution due to their possible transformation during PAH transport from the source to the receptor.

**Figure 4:**
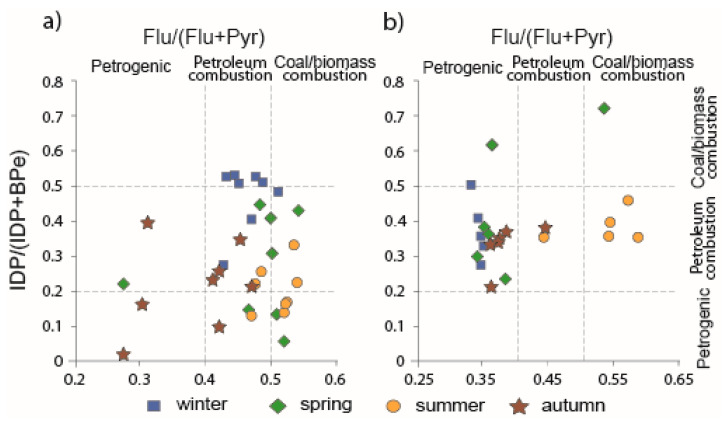
PAHs diagnostic ratios in the surface water of the Partizanskaya River Estuary (**a**) and the Tumen River Estuary (**b**).

In the Partizanskaya River estuary, most of the Flu/(Flu + Pyr) ratio values indicated that PAH originated from the burning of fossil fuels and biomass for the seasons from winter to summer. However, in the autumn, the Flu/(Flu + Pyr) ratio values were relatively separated from the others and shifted towards the range of petrogenic values (0.38 ± 0.08) (Figure 4a). For the IDP/(IDP + BPe) ratio, the highest values were observed in the winter samples, implying coal and biomass burning, while the values of the other samples were lower and showed a mixed pattern of petrogenic and petroleum combustion sources of PAHs. For the Tumen River estuary, the Flu/(Flu + Pyr) ratio showed a clear distinction between the summer samples, where PAHs were from coal and biomass combustion, and the others, where the petrogenic PAHs was detected. The IDP/(IDP + BPe) ratio obtained for all samples suggests petroleum burning (0.39 ± 0.11) (Figure 4b).

**Figure 5:**
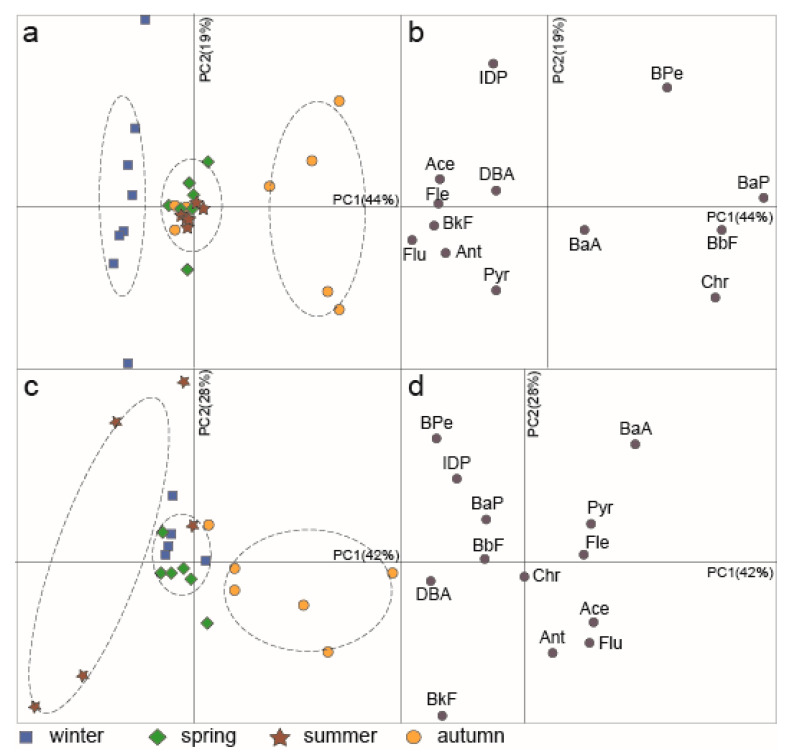
Principal component analysis (PCA) scores for the Partizanskaya River Estuary (**a**) and Tumen River Estuary (**c**). The seasons are differentiated by symbols. The dashed lines outline a grouping by season. The PCA loading plots for PAHs in the Partizanskaya River Estuary (**b**) and Tumen River Estuary (**d**).

Additionally, PCA was used to provide further details on the PAH sources that drive seasonal PAH variation during the year. PC1 represented 44% of the variance for the Partizanskaya River estuary. The score plot shows that samples of this river were isolated into three groups (Figure 5a). The group of winter samples was located on the negative side of the axis and was characterized by a high load of 3-ring PAHs, as well as BkF and Flu (Figure 5b). Autumn samples were grouped on the positive axis, with loading of Chr, BaP, BbF, and BPe. Summer and spring samples were close to the origin and were characterised by moderate loading of the PAHs. For the Tumen River, PC1 presented 42% variance (Figure 5c), and, similar to the Partizanskaya River, three groups of samples were formed. Summer samples located on the negative axis were characterized as having high values of 5–6-ring PAHs and low LMW PAHs (Figure 5d). In contrast, the autumn samples were enriched in 3-ring PAHs and relatively depleted in HMW PAHs. The winter and spring samples were isolated into a mixed cluster near the origin.

PC2, representing 19% and 28% of the variance for the Partizanskaya River and the Tumen River, respectively, did not reveal any seasonal differences between samples and is probably related to the influence of hydrological and meteorological events.

In the Partizanskaya River, enrichment of 3-ring PAHs in the winter samples indicates a petrogenic PAH input, and this contradicts the Flu/(Flu + Pyr) ratio results. This can be attributed to the fact that the diagnostic ratio determines the main PAH source, while minor ones remain hidden [44]. Clustering of the autumn data based on the loading of BPe, which some researchers consider to be a marker of emissions from cars [45], is possibly caused by the PAH input from gasoline engines. This is consistent with some values of Flu/(Flu + Pyr) and IDP/(IDP + BPe) ratios for autumn, indicating the burning of petroleum products. In the Tumen River, the loading of HMW PAHs for the summer samples corresponded well with Flu/(Flu + Pyr) and IDP/(IDP + BPe) ratios, indicating the combustion of fossil fuels and biomass, while the predominance of 3- and 4-ring PAHs in the autumn samples, which suggests a petrogenic origin, was confirmed only by the Flu/(Flu + Pyr) ratio. Taken together, the two isomer ratios and PCA indicate that the major PAH source was the combustion of oil products, coal, and biomass in both rivers. However, there were seasonal changes in the PAH origin while an input of petrogenic PAHs was observed, although it should be considered that the PAH isomeric ratios and PCA results were only partially consistent.

### 3.5. Relationship between PAH Concentration and Salinity

An estuary is a river–ocean transitional zone where freshwater and sea water mixtures occur [46]. In order to understand the major PAH pathway into the estuary, we investigated the relationship between PAHs and salinity. The results showed that in the Tumen River estuary saline water contained three times less PAHs than fresh and brackish water, where the average concentration was 43.3 ng/L (Table 2). The lower PAH concentrations in saline water compared to fresh water have been observed in some estuaries [47,48] and have been attributed to sedimentation or the dilution of relatively clean sea water to polluted freshwater. In contrast, in the Partizanskaya River estuary, the average PAH level in seawater is mostly the same as that in fresh and brackish waters, and the mean concentration is 16.7 ng/L and 18.9 ng/L, respectively. Differences in the distribution of PAH in saline and fresh waters between rivers may be the result of different methods of PAH input to the estuaries. In the Tumen River estuary, the major PAH source is river water, which delivers PAHs from contaminated upstream areas of China and North Korea [31], while in the Partizanskaya River estuary, riverine and coastal waters have common nonpoint PAH sources. In the coastal area adjacent to the Partizanskaya estuary is the town of Nakhodka, which has heavy traffic and several ports with open coal terminals. From there, PAH input probably occurs by atmospheric transport into both river and coastal waters.

Furthermore, depending on the estuary morphology and whether PAHs enter into the estuary with saline or fresh water, the fate of PAH in the estuary ecosystem will vary. Since both estuaries studied are highly stratified [17,18], they are characterized by restricted vertical mixing, with freshwater lens overlying saltier bottom water. Under such conditions, vertical PAH dispersion is inhibited, and, in the case of riverine delivery of pollution to the estuary, PAHs are trapped in the surface freshwater layer [49]. Accordingly, observations in the Tumen River Estuary indicated that higher PAH concentrations were found in surface water. However, in the Partizanskaya River, no vertical PAH gradient was formed, very probably due to both marine and river waters receiving common PAHs from a nonpoint source.

### 3.6. Risk Assessment of PAHs

The results showed that, in both estuaries, RQ_MPCs_ was <1 for each PAH, while the RQ_NCs_ values varied over the year. As shown in Table 3, the largest number of individual PAHs, showing the highest values when RQ_NCs_ > 1, were found for the Tumen River estuary in summer. The eco-toxicological risks of these compounds had reached moderate levels during this period according to the risk classification of individual PAHs (when RQ_MPCs_ < 1; RQ_NCs_ > 1) [19]. It should be noted that in that set, six PAHs (BaA, BbF, BkF, BaP, BPe, IDP) are potentially carcinogenic to mammals, including humans, and one of them, BaP, is listed as a Group 1 carcinogen by the IARC [3]. Furthermore, a moderate level of environmental risk was found mainly for 3- and 4-ring PAHs as well as BbF during other times. The number of PAHs with RQ_NCs_ >1 increased slightly in autumn and winter in both estuaries.

In conclusion, in general the ecotoxicological risk levels were moderate for LMW PAHs and low for HMW PAHs over the year in the estuaries except in the summer in the Tumen river estuary when the RQ values were increased for the majority of PAHs. Nevertheless, it should be taken into account that the mixed effects of multiple contaminants can have a more significant hazard impact on the ecosystem than single-compound exposure. Therefore, the management of PAH pollution in the Tumen Estuary should be considered, especially in summer.

### 3.7. Riverine PAH Mass Flux

The seasonal and annual river TPAH mass flux was estimated based on the daily PAH flux calculated using the Formula (4).

The results presented in Table 4 show that the contribution of the Partizanskaya River into the Sea of Japan amounted to about 0.028 t/year, and that of the Tumen River was two orders of magnitude higher and amounted to 2.5 t/year. The PAH mass flux of some rivers in the world are shown in Table 5 for comparison with the results obtained. In general, the PAH mass flux was lower than that in the rivers presented. However, taking into account the PAH content per unit of volume flow rate (Table 5), these values were low for the Partizanskaya river, while in the Tumen River they were comparable to those in the Rhone River and only lower than those in the Yellow River.

Regarding seasonal variability in PAH riverine input, the maximum TPAH contribution of both rivers, Partizanskaya and Tumen, occurred in summer, which is consistent with the highest TPAH concentrations found for this season in the Tumen River and contrasts with the results for the Partizanskaya River, where the highest concentrations were found in the winter. The summer PAH contribution of the Partizanskaya river amounted to 55% or more of the annual input, and that of the Tumen was 86%. The highest discharge from rivers was observed in summer; consequently, the mass PAH flux into the coastal area of the northwestern Sea of Japan depended on the discharge of rivers rather than the level of PAH pollution. The effect of flood events on PAH mass flux to the marine environment was observed for the Rhone River when 77% of the annual PAH mass flux was discharged to the Mediterranean Sea during the major flood event [50]. The results highlight the need to consider seasonal changes in fluvial PAH flux to the marine environment for modelling of PAH budget and its impact on coastal ecosystems.

## 4. Conclusions

This study examined the seasonal PAH pattern in the estuaries of the Tumen River and Partizanskaya River, which flow into the Northwestern Sea of Japan. On a global scale, the PAH levels were still low in comparison with other water bodies. However, there was PAH enrichment and, consequently, heightened environmental risks for estuarine ecosystems due to seasonal factors, leading to moderate levels of PAHs. A survey of the estuaries showed different trends in the seasonal PAHs variations influenced by humans or natural impacts. Furthermore, the main PAH pathway in the estuaries was also different; for the Tumen River it was polluted riverine water, while PAH pollution of the Partizanskaya River was caused by atmospheric PAH input from nonpoint sources. In addition to assessing the PAH level and environmental risks, we calculated annual and seasonal riverine PAH mass fluxes. The largest PAH contribution of both rivers into the Sea of Japan was in the summer and primarily resulted from the high river discharge.

## Figures and Tables

**Table 1:** Polycyclic aromatic hydrocarbons (PAHs) concentrations in various estuaries in the world during the past decade.

Estuary	PAHs Concentration	Authors
Tumen River, Russia (*n* = 44)	13TPAHs 8.4–159.6 ng/L (33.3 ± 35.1 ng/L) *	This study
	13DPAHs 6.1–66.2 ng/L (17.6 ± 13.8 ng/L) *	
	13PPAHs 2.2–93.3 ng/L (15.7 ± 21.7 ng/L) *	
Partizanskaya River, Russia (*n* = 56)	13TPAHs 10.7–58.6 ng/L (20.3 ± 10.6 ng/L) *	This study
	13DPAHs 6.2–41.3 ng/L (15.4 ± 9.5 ng/L) *	
	13PPAHs 2.9–17.3 ng/L (5.2 ± 2.6 ng/L) *	
Yangtze River, China	16TPAHs 12.9–638.1 ng/L	[26]
Yinma River, China	16TPAHs 175–325 ng/L	[27]
Pearl River, China (*n* = 48)	16TPAHs 133.6–707.7 ng/L 16DPAHs 12.7–160.2 ng/L (87 ± 48 ng/L)	[22]
Sarno River, Italy (*n* = 52)	16DPAHs 12.4–2321.1 ng/L (739 ng/L) 16PPAHs 6.1–778.9 ng/L (254.9 ng/L)	[28]
Urias River, Mexico (*n* = 20)	16TPAHs 9–347 ng/L	[29]
Juilong River, China (*n* = 72)	16TPAHs 17.5–125.9 ng/L	[30]

*n*, number of water samples collected during a year. * Average annual concentration of PAHs during the entire measuring period (for 44 and 56 water samples collected during a year in the Tumen and Partizanskaya estuaries, respectively). DPAHs and PPAHs are the concentrations of PAHs in the dissolved phase and particulate phase, respectively. TPAHs represents the sum of the DPAHs and PPAHs.

**Table 2:** Average annual and seasonal TPAHs concentrations (ng/L) in the fresh and saline waters of the Tumen River Estuary and the Partizanskaya River Estuary.

**Tumen Estuary**					
	**Average Annual**	**Winter**	**Spring**	**Summer**	**Autumn**
Fresh and brackish water	43.3 ± 43.5	19.64 ± 3.76	20.07 ± 5.72	101.54 ± 45.42	29.7 ± 6.96
	(*n* = 29)	(*n* = 6)	(*n* = 8)	(*n* = 8)	(*n* = 5)
Saline water	14.0 ± 9.0	14.31 ± 0.87	7.85 ± 0.89 *	9.69 ± 3.19 *	11.13 ± 1.25 **
	(*n* = 15)	(*n* = 4)	(*n* = 3)	(*n* = 2)	(*n* = 5)
**Partizanskaya Estuary**					
	**Average Annual**	**Winter**	**Spring**	**Summer**	**Autumn**
Fresh and brackish water	18.9 ± 10.5	35.67 ± 10.02	13.21 ± 3.04	11.66 ± 1.00	18.05 ± 3.03
	(*n* = 37)	(*n* = 8)	(*n* = 11)	(*n* = 9)	(*n* = 9)
Saline water	16.7 ± 4.5	-	13.94 ± 3.13	15.36 ± 3.28	20.06 ± 4.53
	(*n* = 19)		(*n* = 5)	(*n* = 7)	(*n* = 7)

Statistically significant differences between fresh and brackish water and saline water are indicated by asterisks (two-sample *t*-tests, * *p* < 0.1; ** *p* < 0.05).

**Table 3:** Mean RQ_NCs_ values for individual PAHs in the estuaries.

	Tumen River Estuary				Partizanskaya River Estuary			
	Winter	Spring	Summer	Autumn	Winter	Spring	Summer	Autumn
Ace	**3.2**	**2.3**	**19.3**	**2.7**	**6.6**	**1.7**	**1.4**	**2.5**
Fle	**8.8**	**7.4**	**21.5**	**7.8**	**19.2**	**5.2**	**5.2**	**7.5**
Ant	**1.2**	0.7	**5.6**	0.7	1.0	0.1	0.1	0.2
Flu	**1.1**	0.9	**7.2**	**1.2**	**2.0**	**1.2**	**1.3**	**1.1**
Pyr	**8.4**	**5.8**	**25.5**	**8.6**	**10.1**	**5.7**	**5.7**	**6.9**
BaA	**10.0**	**5.2**	**26.5**	**10.7**	**6.5**	**2.9**	**2.0**	**4.6**
Chr	0.2	0.1	0.7	0.1	0.3	0.1	0.1	0.6
BbF	**2.4**	**2.3**	**18.6**	**1.3**	**3.1**	0.8	0.6	**3.8**
BkF	0.0	0.1	**1.3**	0.2	0.4	0.0	0.0	0.0
BaP	0.4	0.4	**1.9**	0.4	0.3	0.1	0.1	0.7
DBA	0.1	0.1	0.3	0.2	0.6	0.2	0.1	0.2
BPe	0.5	0.5	**5.7**	0.6	0.9	0.3	0.3	0.9
IDP	0.2	0.2	**2.2**	0.2	0.6	0.1	0.1	0.2

Ace—acenaphthene, Fle—fluorine, Ant—anthracene, Flu—fluoranthene, Pyr—pyrene, BaA—benz[a]anthracene, Chr—chrysene, BbF—benzo[b]fluoranthene, BkF—benzo[k]fluoranthene, BaP—benzo[a]pyrene, DBA—dibenz[a,h]anthracene, BPe—benzo[g,h,i]perylene, IDP—indeno [1,2,3-cd]pyrene.

**Table 4:** TPAHs mass fluxes to the Sea of Japan from the Tumen River and Partizanskaya River and the mean discharge of the rivers.

	Tumen River			Partizanskaya River		
	J, kg	J_mean_, kg	Q, m^3^/s	J, kg	J_mean_, kg	Q, m^3^/s
Winter	1.1–1.8	1.3	8.4	1.3–2.4	1.6	8.3
Spring	289–704	332	475	2.9–4.8	3.6	37.1
Summer	1914–4924	2182	999	12.8–27.8	15.8	47.0
Autumn	10–19	12	67.5	6.0–12.0	7.4	55.1
Annual	2214–5649	2527	390.5	23.0–46.9	28.4	36.9

**Table 5:** PAHs concentrations, river discharge, and fluxes of PAHs from global rivers.

	Discharge, km^3^/yr	PAHs ng/L/N *	PAH Flux, Tons/yr	PAH Per Unit Volume Flow Rate t/km^3^/yr **	Coast	References
Tumen River	6.78 [17]	18.5–88.9/13	2.2–5.6 (mean 2.5)	0.3–0.8	Northwestern Sea of Japan	This study
Partizanskaya River	1.32 [18]	11.7–35/13	0.023–0.047 (mean 0.028)	0.017–0.036		
Pearl River	350	126/15	33.9	0.1	South China Sea	[51]
Yangtze River	980		232	0.24	East China Sea	
Yellow River	57		70.5	1.24	Bohai Sea	
Heilongjiang River	350		30.2	0.09		
Brahmaputra River	140		0.4	0.003	Indian Ocean	
Rhone River	33.8		5.3–33	0.16–0.98	Mediterranean Sea	[52]
Ebro River	6.3		1.3	0.2		
Rivers of Jinhae Bay	-	-/16	0.65 × 10^−4^–0.01 (mean 0.0016)		Jinhae Bay, South-western Sea of Japan	[53]

*—the number of studied PAHs; **—calculated on the basis of the data from the refereed papers in the table.

## Supplementary Materials

The following are available online at https://www.mdpi.com/1660-4601/17/17/6019/s1, Figure S1: Relationship between the mean TPAHs concentration and the daily discharge in the Tumen River estuary (a) and the Partizanskaya River estuary (b), Figure S2: (A)—Relative abundance of DPAHs in the Tumen River (left) and Partizanskaya River (right); (B)—Relative abundance of PPAHs in the Tumen River (left) and Partizanskaya River (right), Table S1: The quality values of NCs and MPCs for studied compounds taken from [19].
