# Supplementary material for: Polycyclic Aromatic Hydrocarbons in the Estuaries of Two Rivers of the Sea of Japan
Source: Int J Environ Res Public Health. 2020 Aug 19;17(17):6019. doi: 10.3390/ijerph17176019 (PMC7503489; doi:10.3390/ijerph17176019)
Supplement: Supplementary file 1 [file ijerph-17-06019-s001.pdf]

Supplementary Materials:

Table S1. The quality values of NCs and MPCs for studied compounds taken from [19].

PAH	$C_{QV(NCs)}$, ng/L	$C_{QV(MPCs)}$, ng/L
Ace	0.7	70
Fle	0.7	70
Ant	0.7	70
Flu	3.0	300
Pyr	0.7	70
BaA	0.1	10
Chr	3.4	340
BbF	0.1	10
BkF	0.4	40
BaP	0.5	50
DBA	0.5	50
BPe	0.3	30
IDP	0.4	40

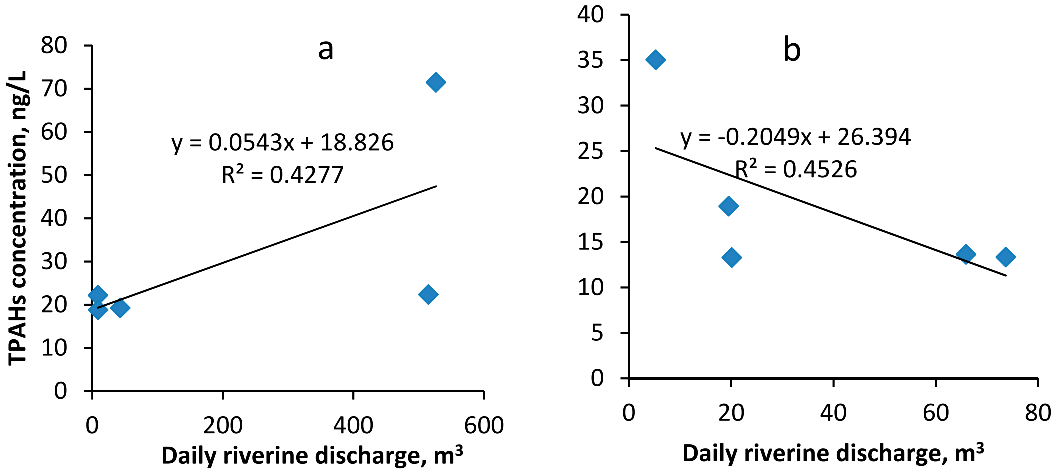

Figure S1. Relationship between the mean TPAHs concentration and the daily discharge in the Tumen River estuary (**a**) and the Partizanskaya River estuary (**b**).

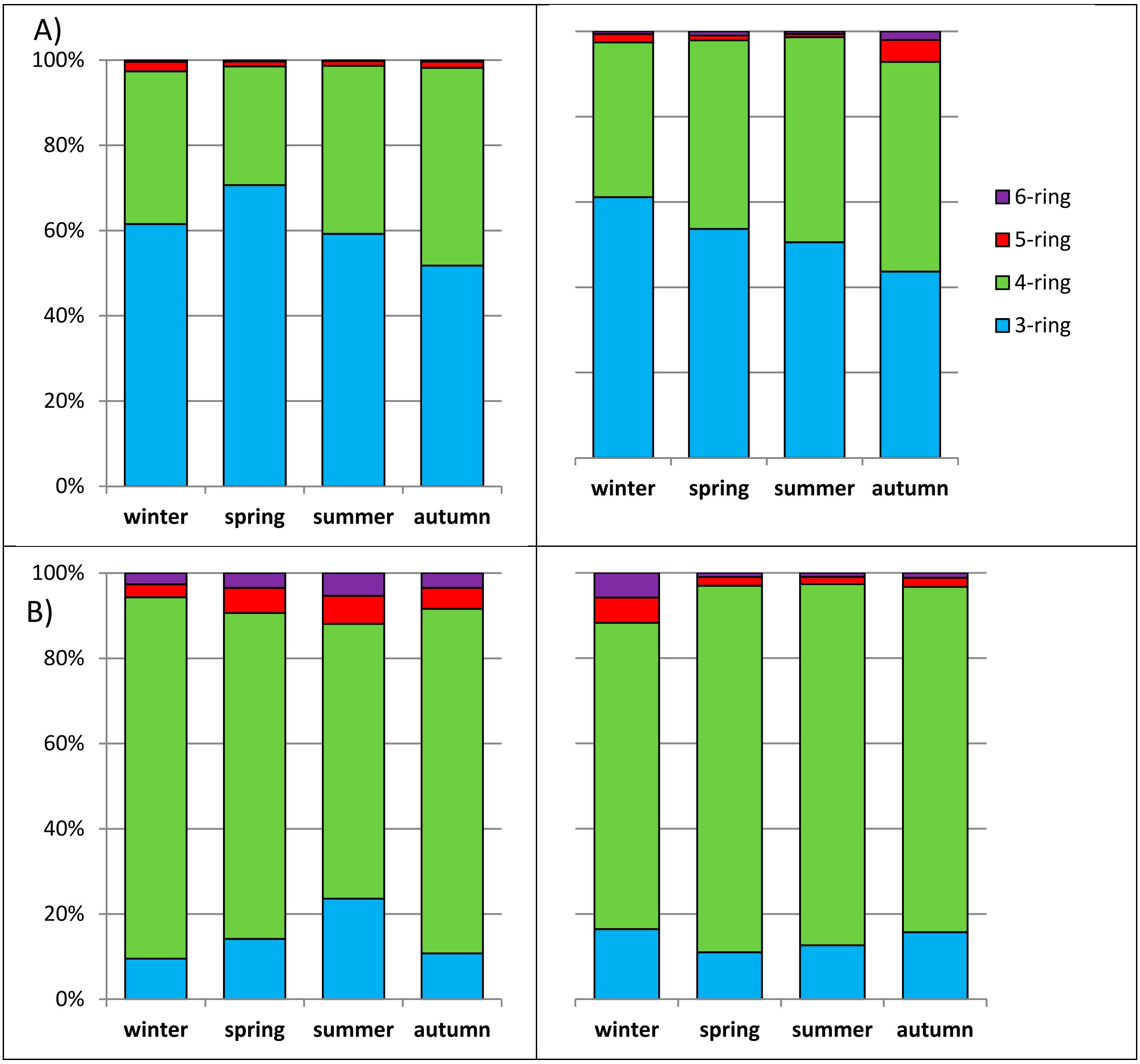

Figure S2. (**A**)—Relative abundance of DPAHs in the Tumen River (left) and Partizanskaya River (right); (**B**)—Relative abundance of PPAHs in the Tumen River (left) and Partizanskaya River (right).
